# ROUNDS Studies: Relation of OUtcomes with Nutrition Despite Severity—Round One: Ultrasound Muscle Measurements in Critically Ill Adult Patients

**DOI:** 10.1155/2018/7142325

**Published:** 2018-04-01

**Authors:** Carlos Alfredo Galindo Martín, Reyna del Carmen Ubeda Zelaya, Enrique Monares Zepeda, Octavio Augusto Lescas Méndez

**Affiliations:** ^1^Nutrition Department, Hospital San Ángel Inn Universidad, Ciudad de Mexico, Mexico; ^2^Critical Care Medicine Resident, Hospital San Ángel Inn Universidad, Ciudad de Mexico, Mexico; ^3^Intensive Care Unit, Hospital San Ángel Inn Universidad, Ciudad de Mexico, Mexico; ^4^Intensive Care Unit, Hospital General de Zona 1, Instituto Mexicano del Seguro Social, Oaxaca de Juárez, Oaxaca, Mexico

## Abstract

Malnutrition (undernutrition) encompasses low intake or uptake, loss of fat mass, and muscle wasting and is associated with worse outcomes. Ultrasound has been introduced in the intensive care unit as a tool to assess muscle mass. The aim of the present study is to explore the relation between initial muscle mass and mortality in adult patients admitted to the intensive care unit. *Methods*. Rectus femoris and vastus intermedius thicknesses were measured by B-mode ultrasound in adult patients at admission, along with demographic characteristics, illness severity, comorbidities, biochemical variables, treatments, and in-hospital mortality as main outcomes. Analysis was made comparing survivors versus nonsurvivors and finally using binary logistic regression with mortality as dependent variable. *Results*. 59 patients were included in the analysis, severity measured by sequential organ failure assessment (SOFA) score was greater in nonsurvivors (17 (7) versus 24 (10) and 3 (1–5) versus 7 (3–10), resp.). Also, muscle thickness was lower in the latter group (1.44 (0.59) cm versus 0.98 (0.3) cm). Logistic regression showed severity by SOFA score as a risk factor and muscle thickness as a protective factor for mortality. *Conclusion*. Muscle mass showed to be a protective factor despite severity of illness; there is much more work to do regarding interventions and monitoring in order to prevent or overcome low muscle mass at admission to the intensive care unit.

## 1. Introduction

For years, the presence of malnutrition (undernutrition) has been related to worse clinical outcomes [1]. These investigations have reached the intensive care unit (ICU) as well [2].

One of the malnutrition definitions correlates with the lack of nutritional intake or uptake that causes changes in the body (lean body mass mainly) composition with impaired clinical outcomes and function [3]. Also, one of the most important nutritional assessments, the Subjective Global Assessment (SGA), considers as main components its structure, the percentage of body weight loss, and the physical exploration regarding the evaluation of the loss of subcutaneous tissue and muscle wasting. Such compartments can be found to be extremely difficult to assess, and to date there is no reliable tool to perform these measures in critically ill patients, even in the early phase of admission to the ICU [4]. Some studies have correlated malnutrition with poor clinical outcomes in critically ill patients relying on scores or screening tests that were generated in noncritically ill patients and also do not account for body composition or use clinical examination, a highly subjective and less exact technique [2, 5–8].

Regarding nutritional assessment in critically ill patients, recent guidelines recommend using the Nutrition Risk in Critically Ill (NUTRIC) and Nutrition Risk Score 2002 (NRS 2002) scores. These tools do not account for body composition; in the case of NRS 2002, it relies on body mass index (BMI) which does not differentiate between muscle, fat, skin, and other tissues. At the same time, guidelines do not recommend using traditional anthropometric or biochemical markers for nutritional evaluation of the critically ill population because they can be altered by fluid overload and can be decreased by inflammatory processes [9]. Other techniques such as bioelectrical impedance can lead to erroneous results influenced by hydroelectrolytic abnormalities normally present in this population [10]. These arguments and recommendations underlie the need for an accessible tool or technique, easy to perform, and not dependent on the information given by the patient or caregivers to assess nutritional status in critically ill patients. The development of such tools could encourage posterior works regarding interventions to overcome malnutrition at the ICU.

The use of imaging techniques has gained importance in the assessment of lean mass in critically ill patients, and the use of computed tomography (CT) has become one of the gold standards. This technique has major problems because it needs the transfer of the patient to a specific area, needs specialized personnel for the study, and is rather expensive, making it not justifiable only as a nutritional assessment tool [11, 12]. Low skeletal muscle mass measured by CT scan has shown negative associations with a wide variety of clinical outcomes such as mortality, length of stay, health-care costs, and discharge disposition. However, as mentioned before, CT scan is not a suitable tool for the daily practice [13–15].

As an alternative, bedside muscle ultrasound has been proposed as an accessible tool in critical care areas, showing good interrater and intrarater correlation, without needing specialized personnel [12, 16, 17]. Multiple techniques of muscular ultrasound have been published in different populations; at present, there is no universal consensus in which technique or threshold should be used [18].

However, ultrasonography is operator dependent and may be susceptible to suboptimal technique and interobserver errors. It also requires that operators adhere to a strict imaging protocol.

The aim of the present (Round One, a part of two studies assessing the effects of malnutrition in critically ill patients) study is to explore a relationship between mortality and muscle mass measured by ultrasound independently of illness severity. We used an internally developed technique for measuring the rectus femoris (RF) and vastus intermedius (VI) at bedside [18].

## 2. Methods

### 2.1. Selection and Initial Variables

A prospective observational study in mixed medical-surgical group of adult critically ill patients admitted to the intensive care unit from April 1, 2016, to March 1, 2017, including every nonpregnant adult (>18 years old) admitted to the unit with a minimum of 48 hours of stay, was carried out. We excluded every patient with neuromuscular diseases, hip fracture, right femur fracture, right thigh burn, any procedure made on the right leg, right lower limb amputation, pregnancy, and palliative care.

Ethical approval was given by the institutional ethics and investigation committee (year 2016/0001).

During the first 24 hours from admission, demographic, biochemical, and clinical data were collected; this included age, gender, stature, height, body mass index, diagnosis of sepsis, Acute Physiology and Chronic Health Evaluation II (APACHE II), Sequential Organ Failure Assessment score (SOFA score), Charlson Comorbidity Index (CCI), Nutrition Risk in the Critically Ill score (NUTRIC score), and biochemical variables such as glucose, creatinine, urea, blood urinary nitrogen, complete electrolytes (Na, K, Cl, Ca, Mg and P), C-reactive protein (CRP), procalcitonin (PCT), lactate, bicarbonate, and serum pH (from a venous blood gas analysis).

**Figure 1:**
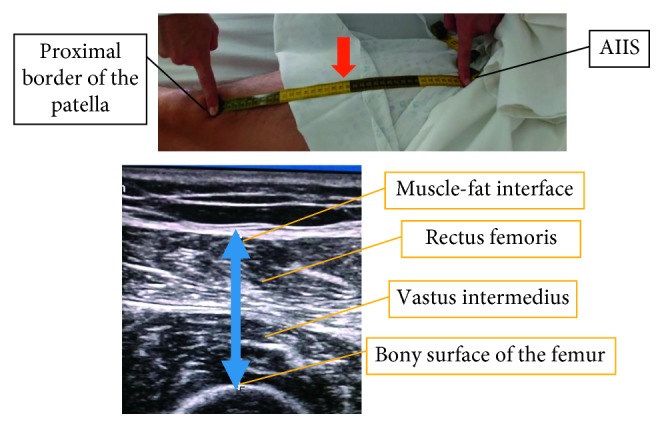
Ultrasound measurement technique. AIIS: anterior inferior iliac spine; red arrow: midpoint; blue arrow: measured distance (thickness).

During the first 48 hours, measurement of the thickness of RF and VI muscles by ultrasound was performed according to the following technique (developed internally by this investigation team, Figure 1):

- Ensure complete supine position of the patient, with the head of the bed at 30–45°
- Place the right leg extended and with the toes pointing at the ceiling
- Use a nonstretchable fiberglass measuring tape and measure the distance from the anterior inferior iliac spine (AIIS) to the midpoint of the proximal border of the patella
- Locate the midpoint of the previous distance
- Position the linear transducer at 13 MHz in B-mode perpendicular to the measured line and over the midpoint, forming a 90° angle
- Locate the femur using the ultrasound controls without tilting or displacing the transducer from the initial point and apply maximum pressure
- Freeze the image
- Mark the distance between the bony surface of the femur and the muscle-fat interface using a vertical line in order to measure the thickness of both muscles excluding fat mass, skin, and bone. Start with the midpoint or highest point of the femur surface and trace a vertical line upwards
- Repeat the technique, and if the difference between the measures is greater than 0.1 cm (randomly assigned value), perform a third measure
- Calculate the mean thickness (round to 2 decimals). The calculations were performed as follows: (measure 1 + measure 2)/2 or (measure 1 + measure 2 + measure 3)/3, as necessary

We obtained only one value of muscle thickness for each patient.

Measurements were performed by a single person after a one-week period of practice in healthy adults.

### 2.2. Follow-Up

Data regarding treatments were collected, including the need for renal replacement therapy, insulin, vasoactive drugs, invasive mechanical ventilation, steroids, and sedation during any day of the first 7 days of intensive care unit stay. During this same stage, intensive care unit length of stay (LOS) and general mortality (anytime during hospitalization) were collected. Follow-up after hospitalization was not performed.

### 2.3. Outcomes

Primary outcome was in-hospital mortality, and the population was divided according to their discharge status (dead/alive), and the secondary outcome was ICU LOS. In-hospital mortality was chosen as the primary outcome in order to assess short-term effects of muscle wasting.

### 2.4. Statistical Analysis

For the statistical analysis, quantitative data were tested with the Kolmorogov–Smirnov test to assess the type of distribution. Normally distributed variables were presented as mean (standard deviation) and nonnormally distributed variables as median (interquartile range: 25th percentile–75th percentile). For comparison between groups, *t-* test for independent group or Mann–Whitney *U* test was performed in normally or nonnormally distributed variables, respectively. Qualitative variables were presented as frequency and percentage (*n* (%)), and chi-square or Fisher's exact test was used for comparisons as required.

Finally, a binary logistic regression analysis was performed using the discharge status (alive versus dead) as a dependent variable, and a final model was obtained by including as independent variables those with statistical significance in bivariate analysis. Accuracy of the model was obtained, and the model which showed the highest accuracy in which other variables did not improve and showed to be the most real-life operative model without collinearity was selected as the final result. Predicted mortality was calculated in every patient according to the model, and a receiver operating characteristics curve (ROC curve) was obtained to assess the performance of the model to detect mortality. Every *p* value less than 0.05 was considered significant.

## 3. Results

Seventy-two patients were eligible for the study, and reasons to exclude patients were as follows: 3 were transferred to other hospitals, 5 stayed less than 48 hours, 2 patients or their caregivers refused the study, and 3 patients were measured beyond 48 hours. A total of 59 patients were included in the final analysis demographic, and initial characteristics are shown in Table 1. Overall, 12 patients died during hospital stay (20.3%), and 47 patients survived (79.7%).

Those patients who died had higher severity scores in comparison to those who survived, there was no difference between groups regarding age, gender, BMI, sepsis diagnosis, CCI, and NUTRIC score.

**Figure 2:**
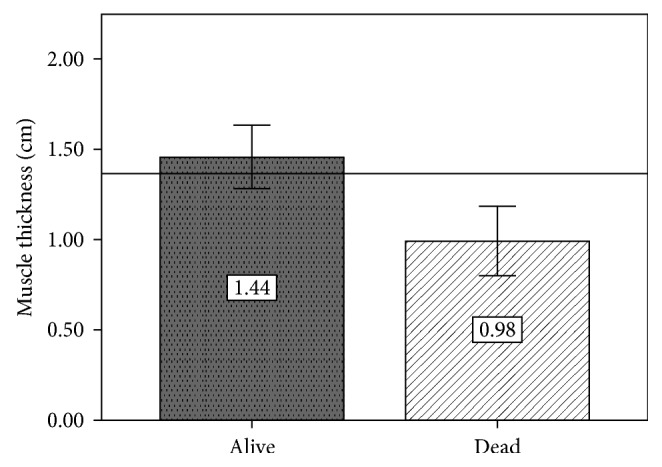
Mean muscle thickness at admission. Horizontal line: general mean (1.35 cm); *p* < 0.05 indicates difference between groups. Measures were taken in less than 48 hours from admission to the ICU.

In relation to muscle thickness (RF and VI) at admission measured by ultrasound, those patients who survived showed greater thickness compared to the nonsurvivors group (Figure 2). 39 patients (83%) in the alive group were measured during the first 24 hours and 8 during the next 24 hours; in the nonsurvivors group, 9 patients (75%) were measured during the first 24 hours and 3 during the next 24 hours.

Regarding biochemical variables, lactate levels were higher in the group of nonsurvivors, without other differences in this group of variables (Table 2).

No difference was seen in ICU LOS, renal replacement therapy, and use of vasoactive drugs and steroids (Table 3). More patients in the nonsurvivors group presented high nutritional risk (NUTRIC score > 4 points), used insulin therapy, and were ventilated and sedated. All patients in the survivors group had mobility with a significant difference between groups (*p* < 0.05).

**Figure 3:**
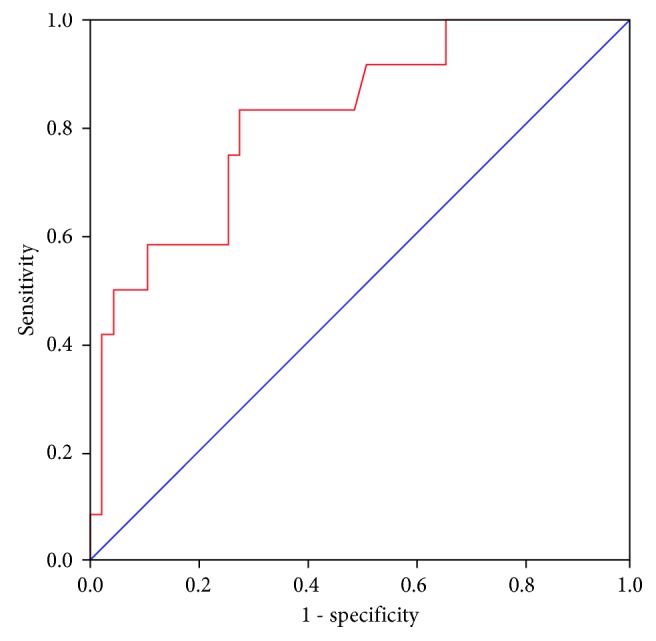
Receiver-operating characteristic curve of predicted mortality by the binary logistic regression model. Blue line: 50% of area under the curve reference.

In the final model of binary logistic regression with discharge status as the dependent variable, only the SOFA score and the muscle thickness at admission were included in the model, given that other variables showed no improvement in the accuracy or were not significant in bivariate analysis (Tables 4 and 5, Figure 3). Age, gender, and sepsis were not significant at *p* > 0.05, so these variables do not have a correlation with mortality in this group. The model did not change even when gender or age was included in separate models, sustaining an accuracy of 86% (data not shown). Other variables such as IMV, NUTRIC, APACHE II, lactate, and insulin use did not improve the accuracy of the model or introduced collinearity (NUTRIC score uses SOFA and APACHE II; finally, both APACHE II and SOFA are severity scores).

Final model obtained 83% of sensitivity and 87% of specificity.

## 4. Discussion

In the present study, we found an association between muscle mass at admission proving to be an independent protective factor for mortality despite severity according to the SOFA score, which showed to be a risk factor (as all of us already know).

Low muscle mass can be observed more frequently in the elderly population. Moisey et al. retrospectively analyzed CT scans in order to measure muscle mass in elderly patients (>65 years old, *n*=149) admitted to the ICU (level 1 trauma center), and quantification of muscle was obtained from images at L3 level. To normalize values, they adjusted the muscle mass by height in square meters (cm^2^/m^2^), and sarcopenia was defined using previously cutoff values for men and women. Initially, BMI did not show a consistent trend or relation with the muscle status, and sarcopenic patients were older than their counterparts. In terms of mortality, more than twice the sarcopenic patients died in the hospital compared with nonsarcopenic patients. After adjusting for age, sex, and severity, the muscle mass was associated with mortality as a protective factor. Neither BMI nor albumin was associated with the primary outcome of mortality [13].

Weijs et al. retrospectively studied 240 critically ill patients analyzing the muscle mass by CT scans at the level of L3 made in the period of time of −1 to 4 days from admission to the ICU. Those patients with low muscle area, defined with a cutoff point of muscle mass to predict in-hospital mortality for men and women separately, were older, had higher APACHE II score, lower weight, and longer hospital stay, and were most likely to be discharged to a nursing home. In a regression analysis, they found that low skeletal muscle area (in cm^2^) was an independent risk factor for mortality, adjusted for age, gender, BMI, diagnosis, and APACHE II (severity) [14].

Fuchs et al. also analyzed L3 CT scans in order to measure muscle mass in 231 adult critically ill patients that required mechanical ventilation. CT scans were performed within 5 days of extubation in order to explore the relation between the muscle mass and post-extubation outcomes. Muscle mass values were adjusted for height (cm^2^/m^2^) and gender using preestablished values for correction. Skeletal muscle mass showed to be an independent protective factor for post-extubation pneumonia, adverse discharge disposition, and 30-day mortality. As a secondary outcome, muscle mass showed no correlation with reintubation and LOS but was associated with hospital costs. All analyses were adjusted for age, APACHE II, and CCI. This is the first study that correlates the muscle mass with hospital costs and is in agreement with previous studies in terms of mortality [15].

The previous studies show consistent results demonstrating that muscle mass can be useful to predict adverse outcomes, given that low muscularity correlates with poorer outcomes despite severity of illness or injury. Unfortunately, these results rely on CT scan measurements, and its use in the daily practice to perform a nutritional assessment of critically ill patients is not realistic and not cost-effective and could increase the complication rates and workload. Those are the reasons why we tried to use ultrasound, given that is an accessible tool at the ICU because it is used in many other procedures (central line placement, neurological monitoring, and hemodynamic monitoring). Also in the present study, we found analogous results regarding the correlation between the muscle mass and mortality.

Mueller et al. conducted a prospective study in 102 surgical critically ill adult patients with at least 72 hours of stay in the unit to explore the correlation between sarcopenia and outcomes in this population. Muscle ultrasound of rectus femoris cross-sectional area (RF-CSA) using minimum pressure and adjusting for sex preestablished values was performed in every patient at admission. Sarcopenia was defined with a cutoff value of muscle mass (RF-CSA in cm^2^) that detects frailty defined by the Youden Index (5.2 cm^2^, area under the curve: 0.75, 73% sensitivity, and 69% specificity). Those patients with sarcopenia were older and had higher burden of comorbidities and greater muscle weakness (by the Medical Research Council Scale of 12 different muscles), even though 18% of the sarcopenic patients were less than 50 years old, showing that this condition is not only present in the elderly population. As sarcopenic patients had higher APACHE II score at admission, all of the patients who died were sarcopenic, and the presence of this condition was found to be an independent predictor of adverse discharge disposition (dead or skilled nursing facility) adjusted for age, creatinine, hemoglobin, severity, comorbidities, and Glasgow coma score [19] In concordance with the present study, the use of muscle ultrasound shows its utility as a risk assessment at admission to the ICU. However, in the present study, muscle mass was not adjusted for gender because of the lack of difference between groups in terms of sex, and we used crude numbers to perform our analysis. Finally, the techniques of measurement are completely different, given that we measured the thickness of both muscles using maximum pressure and not the area of a single muscle. These differences do not rule out the correlation between muscle and outcomes but just show the lack of agreement among different techniques.

Sabatino et al. studied the difference between measurements of muscle (rectus femoris and vastus intermedius at 1/2 and 1/3 of the distance between the patella and the anterior superior iliac spine, and minimum pressure) by ultrasound before and after hemodialysis in critically ill patients with acute kidney injury as a secondary outcome. They found no difference between measurements, even when they analyzed only those patients with negative weight balance after hemodialysis [20]. This supports the use of ultrasound in critically ill patients even when there are changes in fluid balance, showing that there is no influence of fluid status in muscle thickness measured by ultrasound.

The following studies found significant results but using less exact techniques.

Bector et al. used SGA to assess 57 critically ill medical patients, showing greater mortality in those patients with an SGA of B (moderately malnourished) and SGA of C (severely malnourished) compared with those with an SGA of A (well nourished), and also found greater APACHE II scores in nonsurvivors compared with survivors, without any difference in severity when compared with the nutritional status [5]. One of the main weaknesses of this study is that SGA is difficult to perform in nonconscious, noncooperative, bedridden, and with fluid overload ICU patients. Given this, the SGA is found to be rather inexact in determining nutritional status in these populations. It is important to remark that when the SGA was developed, the original team found that the clinician was more influenced by the components of weight loss, loss of subcutaneous tissue, and muscle wasting (quadriceps and deltoids mainly) to determine whether a patient was undernourished [4]. In agreement with our study, the muscle mass was evaluated by ultrasound (quadriceps muscles too), the only variable of the SGA we can obtain objectively, whereas the other variables are difficult-to-impossible to obtain and are with low reliability. Inversely, we found muscle mass as a protective factor that means, with greater muscle thickness, less probability for mortality.

Multiple studies in the Brigham and Women's Hospital have shown the effects of malnutrition in critically ill populations. The nutritional assessment of the three studies referenced here use four main components to determine nutritional status in every patient studied, namely, (1) weight loss, (2) reduced intake, and signs of malnutrition such as (3) muscle wasting and/or (4) fat-tissue loss even without anthropometric foundation. These studies have found worse clinical outcomes in emergency surgery patients and mixed ICU populations with malnutrition and even worse outcomes regarding mortality and readmission rates one year after discharge, even when adjusted to number of organ failures [6–8]. Nonobjective measures of the muscle mass were performed, and rather subjective assessment was used. We can agree that nutritional status is an independent risk factor for mortality when adjusted to organ failures, the same thing that the SOFA score takes into account and is used as a severity score. In this study, follow-up to capture total LOS and post-discharge mortality or other outcomes such as quality of life and readmission was not performed, which could give more useful insights and value to this investigation.

Biochemical assessment of the muscle status has also been investigated in patients with prolonged mechanical ventilation. Using creatinine to high index (CHI) as a muscle status marker, Data et al. showed that low (CHI) was found to be a predictor of weaning failure and mortality in a population of 167 patients [21]. This supports the importance of low muscle mass during chronic inflammation or long LOS; given that multiple undernourished patients return to the hospital, detecting those patients with previous severe muscle wasting is important to integrate a more aggressive multidisciplinary plan.

The main weaknesses of the present study are the absence of long-term follow-up, small sample size, and no physical function and quality-of-life assessment. Also, there is no universal technique or cutoff point of muscle thickness to consider “low” or “high.”

The strengths are the prospective nature of the study, the use of a bedside technique for real-time and real-life measurements (that can be performed in the daily practice), and the high performance of the constructed model to predict in-hospital mortality.

Since the beginning, lean body mass has been considered one of the most important prognostic factors, relating malnutrition and/or low muscular mass with worse outcomes. New technologies are emerging, and the bedside evaluation of muscle mass in ICU population has been progressing giving us the opportunity to overcome the usual difficulties in this scenario.

## 5. Conclusion

In the present study, RF and VI thickness measured by ultrasound as a surrogate of muscle mass was found to be an independent protective factor for mortality despite the illness severity in critically ill adult patients. Studies with more patients, centers, long-term follow-up (considering physical function and quality of life), and a wider variety of measurements in each patient are required in the future.

## Figures and Tables

**Table 1:** Demographic and initial characteristics.

	Alive (*n*=47)	Nonsurvivors (*n*=12)	Total (*n*=59)
Age (years)^#^	59 (43–74)	73 (54–84)	63 (44–75)
Male, *n* (%)	15 (31.9)	5 (41.7)	20 (33.9)
BMI (kg/m^2^)^&^	25.67 (5.81)	26.21 (6.61)	26.19 (5.08)
Sepsis diagnosis, *n* (%)	18 (38.3)	6 (50.0)	24 (40.7)
APACHE II (points)^&^	17 (7)^∗^	24 (10)^∗^	19 (8)
SOFA (points)^#^	3 (1–5)^∗^	7 (3–10)^∗^	4 (1–6)
CCI (points)^#^	2 (0–2)	2 (0–2)	2 (0–2)
NUTRIC (points)^#^	3 (2–5)	6 (2–7)	3 (2–5)

BMI: body mass index; APACHE II: Acute Physiology and Chronic Health Evaluation II; SOFA: Sequential Organ Failure Assessment score; CCI: Charlson Comorbidity Index; NUTRIC: Nutrition Risk in the Critically Ill score. ^∗^*p* < 0.05 indicates difference between groups; ^#^median (interquartile range); ^&^mean (standard deviation).

**Table 2:** Biochemical variables at admission.

	Alive (*n*=47)	Nonsurvivors (*n*=12)	Total (*n*=59)
Na (mEq/L)^&^	137 (7)	137 (9)	137 (7)
K (mEq/L)^&^	4.2 (0.9)	4.2 (0.6)	4.2 (0.9)
Cl (mEq/L)^&^	100.39 (7.35)	97.5 (11.36)	99.79 (8.30)
Ca (mg/dL)^&^	7.82 (0.82)	8.16 (0.60)	7.90 (0.80)
P (mg/dL)^&^	4.20 (1.32)	4.24 (1.20)	4.2 (1.29)
Mg (mg/dL)^#^	1.9 (1.50–2.00)	1.9 (1.5–2.05)	1.9 (1.5–2.00)
Lactate (mmol/L)^#^	1.55 (1.10–2.30)^∗^	3.15 (2.30–4.50)^∗^	1.75 (1.2–2.9)
Bicarbonate (mEq/L)^#^	21.50 (19.00–23.00)	21 (17.95–23.50)	21.00 (19.00–23.00)
pH^#^	7.35 (7.3–7.4)	7.36 (7.25–7.4)	7.35 (7.3–7.4)
CRP (mg/dL)	6.73 (2.02–12.18)	10.60 (7.10–22.00)	7.99 (2.95–12.95)
PCT (mg/L)^#^	0.5 (0.20–1.90)	0.41 (0.14–1.13)	0.5 (0.19–1.67)
Glucose (mg/dL)^#^	130 (104–170)	158 (140–221)	138 (111–191)
Creatinine (mg/dL)^#^	0.78 (0.55–1.93)	0.74 (0.64–1.38)	0.78 (0.57–1.69)
Blood urinary nitrogen (mg/dL)^#^	21.00 (13.00–37.00)	20 (13.37–17.12)	21.00 (13.00–37.00)
Urea (mg/dL)^#^	40.00 (27.00–70.00)	40.00 (21.50–78.50)	40.00 (26.00–74.00)

PCT: procalcitonin, CRP: C-reactive protein. ^∗^*p* < 0.05 indicates difference between groups; ^#^median (interquartile range); ^&^mean (standard deviation).

**Table 3:** Length of stay, therapies, and qualitative variables.

	Alive (*n*=12)	Nonsurvivors (*n*=47)	Total (*n*=59)
LOS (days), median (interquartile range)	4 (3–7)	4 (3–8)	4 (3–7)
IMV, *n* (%)	13 (27.7)^∗^	10 (83.3)^∗^	23 (39.0)
Mobility, *n* (%)	47 (100)^∗^	8 (66.7)^∗^	55 (93.2)
Sedation, *n* (%)	15 (31.9)^∗^	10 (83.3)^∗^	25 (42.4)
Steroids, *n* (%)	11 (23.4)	1 (8.3)	12 (20.3)
Norepinephrine, *n* (%)	10 (21.3)	6 (50.0)	16 (27.1)
Vasopressin, *n* (%)	16 (34.0)	8 (66.7)	24 (40.7)
Insulin, *n* (%)	18 (38.3)^∗^	9 (75.0)^∗^	27 (45.8)
Hemodialysis, *n* (%)	6 (12.8)	0 (0)	6 (10.2)
High nutritional risk, *n* (%)	12 (25.5)^∗^	7 (58.3)^∗^	19 (32.2)

LOS: length of stay; IMV: invasive mechanical ventilation; high nutritional risk: NUTRIC ≥ 5. ^∗^*p* < 0.05 indicates difference between groups.

**Table 4:** Binary logistic regression: discharge status (in-hospital mortality) as dependent variable.

Variables	*B*	*p*	Exp (*B*)	95% confidence interval	
				Lower limit	Upper limit
USG1	−2.20	0.02	0.11	0.02	0.74
SOFA	0.26	0.02	1.29	1.05	1.60

USG1: muscle thickness at admission; SOFA: Sequential Organ Failure Assessment score; percent of correctness (accuracy) 86.4%.

**Table 5:** Area under the curve for the predicted mortality assigned by binary logistic regression model.

AUC	*p*	95% confidence interval	
		Lower limit	Upper limit
0.82	<0.001	0.69	0.95

AUC: area under the curve.
